# Gut microbiota and metabolic alterations in participants with flatulence identify *Faecalibacterium prausnitzii* as a key microbial target for clinical intervention

**DOI:** 10.1080/19490976.2026.2728332

**Published:** 2026-09-03

**Authors:** Ruimin Chen, Chuan Zhang, Shikai Yan, Chengcheng Zhang, Yilin Ren, Gang Wang, Shumao Cui, Wenwei Lu, Bo Yang, Xiaomei Lyu, Peijun Tian, Jianxin Zhao, Fengwei Tian, Yuzheng Xue, Qixiao Zhai

**Affiliations:** a State Key Laboratory of Food Science and resources, Jiangnan University, Wuxi, Jiangsu, People's Republic of China; b School of Food Science and Technology, Jiangnan University, Wuxi, Jiangsu, People's Republic of China; c Department of Gastroenterology, Affiliated Hospital of Jiangnan University, Wuxi, Jiangsu Province, People's Republic of China; d National Engineering Research Center for Functional Food, Jiangnan University, Wuxi, Jiangsu, People's Republic of China

**Keywords:** Flatulence, gut microbiota, clinical trial, probiotics, gas

## Abstract

Flatulence is closely associated with gut dysbiosis, yet the characteristic microbial signatures, metabolic alterations, and actionable intervention targets remain unclear. This limited mechanistic understanding has hindered the development of precise microbiota-based strategies for managing flatulence. Here, we found that participants with flatulence exhibited marked shifts in gut microbial functions and fecal metabolic profiles compared with healthy controls, characterized by enhanced abnormal fermentation, enrichment of oxidative stress-related functions, elevated low-grade inflammatory signatures, and reduced anti-inflammatory and mucosal-protective metabolic features. *Faecalibacterium prausnitzii* was significantly negatively associated with the high-gas-producing phenotype. *In vitro* replenishment experiments further validated the role of *F. prausnitzii* in reducing gas production, promoting butyrate generation, and remodeling butyrate-associated microbial communities. Based on microbial interaction analysis, we identified *Bifidobacterium longum* CCFM1319 as a candidate strain for targeting *F. prausnitzii*. In a double-blind, randomized, placebo-controlled clinical trial, supplementation with *B. longum* CCFM1319 significantly increased intestinal *F. prausnitzii* abundance and improved flatulence-related symptoms. Collectively, these findings reveal the microbiota and metabolic dysbiosis underlying flatulence, highlight the key regulatory role of *F. prausnitzii*, and lays the foundation for targeted microbiota-based intervention strategies for flatulence.

## Introduction

In the healthy human intestine, various gases are present, including hydrogen (H₂), methane (CH₄), and carbon dioxide (CO₂), as well as trace gases such as hydrogen sulfide (H₂S), nitric oxide (NO), and sulfur-containing compounds. These gases can be expelled through belching, exhalation, or anal gas evacuation. Anal gas evacuation is generally a normal physiological phenomenon^1^; however, abnormally frequent gas passage, particularly when accompanied by unpleasant odor or gastrointestinal discomfort, may indicate an underlying health problem.^2^ Epidemiological studies have shown that flatulence is highly prevalent, affecting approximately 16%–31% of the general population and up to 66%–90% of patients with irritable bowel syndrome (IBS).^3-5^ Excessive flatulence not only causes social embarrassment but also negatively affects intestinal health, quality of life, and emotional well-being.

Intestinal gas is mainly derived from microbial fermentation of undigested dietary substrates and endogenous compounds. However, gut microbiota composition varies substantially among individuals,^6^ which may influence substrate utilization, gas production, and gas composition. Gut dysbiosis, including small intestinal bacterial overgrowth, has been associated with abdominal distension, flatulence, and bloating. Ringel-Kulka et al. reported that members of Ruminococcaceae and Eubacteriaceae were significantly increased in IBS participants with flatulence symptoms.^7^ Manichanh et al. further showed that, after ingestion of a flatulogenic diet, participants with flatulence displayed greater instability in gut microbiota composition than healthy controls. In addition, taxa such as *Bacteroides fragilis* and *Bilophila wadsworthia* were associated with the frequency or volume of gas evacuation. These findings suggest that flatulence is not merely caused by increased availability of fermentable substrates, but is closely related to specific microbial community structures, fermentative functions, and the host intestinal microenvironment.^2^

Although gut dysbiosis is considered an important driver of flatulence and related functional gastrointestinal discomfort, the key microbial taxa and abnormal metabolic mechanisms involved remain unclear, leaving current microbiota-based interventions without well-defined targets. Probiotics, prebiotics, and synbiotics have been used to alleviate functional gastrointestinal symptoms, and some *Lactobacillus*, *Bifidobacterium*, multi-strain probiotics, galacto-oligosaccharides, and synbiotic formulations have been shown to improve flatulence, bloating, or borborygmi scores.^8-14^ However, other studies have reported no significant benefit, or even worsening of gas-related discomfort.^15-20^ In particular, prebiotics can serve as fermentable substrates for the colonic microbiota and may increase the production of H₂, CO₂, or CH₄ while promoting short-chain fatty acid generation. Therefore, identifying key flatulence-associated microbes and clarifying their mechanisms of action are essential for developing more precise and stable microbiota-based intervention strategies.

This study aimed to characterize the microbial structure, functional features, and metabolic profiles of participants with flatulence, identify key microbial targets associated with the high-gas-producing phenotype, and explore a precision intervention strategy based on microbial interactions. We compared gut microbiota composition, functional characteristics, and gas-producing capacity between individuals with flatulence and healthy controls, identified *F. prausnitzii* as a potential protective target, and validated its effects on gas production and butyrate-associated microbiota remodeling using *in vitro* replenishment experiments. Finally, we screened *Bifidobacterium longum* CCFM1319 and validated its ability to increase intestinal *F. prausnitzii* abundance and improve flatulence symptoms through a clinical intervention. Overall, this study proposes a precision microbiota-based intervention strategy that targets *F. prausnitzii* through modulation by *B. longum*.

## Materials and methods

### Experimental strains and culture conditions

The *Faecalibacterium prausnitzii* strains A2-165 and CCFM1204 and the *Bifidobacterium longum* strains FGXBM14M6, FAHMAS43M10, CCFM1112, FHeJZ28M10, FGDGZ14M5, CCFM1319, FGDLZ8M1, FGDL28M1, FJSWXJ39M4, and FSDTA2M6 were from the Culture Collection of Food Biotechnology Center, Jiangnan University, and were all isolated from feces of healthy human. Four types of media were prepared according to previously published protocols: gut microbiota medium (GMM)^21^ and simulated colonic chyme medium (SM)^22^ were used for *in vitro* fermentation experiments, M2GSC broth^23^ was used for the cultivation of *F. prausnitzii*, and MRS broth was used for the cultivation of *B. longum*. All strains were cultured in an anaerobic chamber at 37 °C.

### Participants and clinical data details

#### Cross-sectional observational study

Prior to the start of the study, all participants were asked to complete a flatulence symptom questionnaire under the professional guidance of a gastroenterologist. The questionnaire was adapted from the study by Manichanh et al. published in *Gut* and assessed the following parameters: (a) number of bowel movements and stool form using the Bristol Stool Form Scale; (b) subjective sensations of flatulence (defined as anal gas evacuation), bloating (pressure/filling), distention (increased waist circumference), abdominal distention and borborygmi, using the corresponding 0−10 analog scale; and (c) digestive tract comfort, using a 10-point scale ranging from +5 (satisfactory) to −5 (unsatisfactory).^2^ Before questionnaire completion, the gastroenterologist explained the definitions of each symptom and the scoring criteria to the participants, with particular emphasis on the scoring of anal gas evacuation frequency, to minimize inter-individual differences in the interpretation of the scale. The exclusion criteria included major bowel disease (intestinal obstruction, intussusception, colon cancer, colitis), respiratory disease or infection; antibiotic use in the previous 2 months; probiotic habit and use of probiotic products or food containing probiotics in the previous 1 month. After applying these exclusion criteria, participants with an anal gas evacuation score ≥5 were assigned to the flatulence group, whereas age- and sex-matched participants with no obvious flatulence-related symptoms and an anal gas evacuation score below this inclusion threshold were assigned to the healthy control group. After enrollment, participants in the flatulence group were asked to provide three fecal samples corresponding to the pre-, during-, and post-flatulence, whereas healthy controls provided one fecal sample. The procedure details are shown in Figure S1. The study was approved by the Medical Ethics Committee of Jiangnan University (JNU20220606IRB04) and was registered with the China Clinical Trials Registry (ChiCTR2300073331). The protocol followed the ethical principles outlined in the Declaration of Helsinki. Before enrollment, all participants provided written informed consent.

#### *B. longum* CCFM1319 intervention trial

A randomized, double-blind, placebo-controlled clinical intervention trial was conducted to evaluate the effects of *B. longum* CCFM1319 on flatulence and *F. prausnitzii* abundance. Participants were screened using a flatulence symptom questionnaire, and the inclusion and exclusion criteria were consistent with those used in the cross-sectional observational study described above. Eligible participants were allocated to either the CCFM1319 group or the placebo group for a 28-day intervention according to a random-number sequence. The treatment assignment was concealed from both the participants and the researchers performing symptom evaluation or sample analysis. *B. longum* CCFM1319 was anaerobically cultured at 37 °C and spray-dried into a ready-to-consume probiotic powder by Yangzhou Jiangda Shisheng Yisheng Food Co., Ltd. (Yangzhou, China). The probiotic product was supplied in 2-g sachets and maintained a viable count of >10^9^ CFU per sachet throughout the intervention period, as confirmed by the stability test shown in Figure S7a. The placebo consisted of maltodextrin and was matched to the probiotic powder in appearance. Participants in the CCFM1319 group received the probiotic powder, whereas those in the placebo group received the placebo powder. Participants were instructed to consume one sachet twice daily for 28 days. Adherence and safety were monitored by telephone or WeChat follow-up during the intervention, during which sachet intake, missed doses, and any discomfort or adverse events were recorded. The procedure details are shown in Figure S2. During the intervention period, participants were instructed to maintain their usual diet and lifestyle and to avoid additional intake of probiotics, prebiotics, synbiotics, antibiotics, or any products that could affect the gut microbiota. Fecal samples were collected before intervention (D0) and after intervention (D28). Changes in flatulence-related symptoms, including flatulence, bloating, abdominal distension, borborygmi, and gastrointestinal comfort, were assessed using the flatulence symptom questionnaire. Fecal samples were stored at low temperature immediately after collection and used for subsequent gut microbiota analysis and absolute quantification of target bacteria. The study was approved by the Medical Ethics Committee of Jiangnan University (JNU20220606IRB04) and was registered with the China Clinical Trials Registry (ChiCTR2300073331).

### Fecal sample collection

#### Cross-sectional observational study and *B. longum* CCFM1319 intervention trial

For the cross-sectional observational study and the *B. longum* CCFM1319 intervention trial, participants were instructed to self-collect fecal samples at home using fecal collection tubes according to the provided instructions. After collection, the samples were immediately placed in a container with a cold pack and delivered to the laboratory within 2 h after defecation. Upon receipt, fecal samples were immediately frozen and stored at −80 °C until subsequent gut microbiota analysis, fecal metabolite measurements, and absolute quantification of target bacteria.

#### *In vitro* fermentation

Fresh fecal samples used for in vitro fermentation experiments were obtained from participants recruited in the cross-sectional observational study. The samples were collected in sterile fecal collection tubes and delivered to the laboratory within 30 min after defecation. The samples were not frozen before fermentation and were processed immediately upon receipt under anaerobic conditions.

### DNA extraction

Upon receipt, fecal samples were immediately frozen and stored at −80 °C. DNA was extracted from samples using the FastDNA Spin Kit (MP Biomedicals Ltd., Santa Ana, CA, USA) according to the Earth Microbiome Project (EMP) standard protocol. The V3–V4 hypervariable region of the 16S rRNA gene was amplified by PCR using extracted DNA as the template and the primer pair 341F (5′-CCTAYGGGRBGCASCAG-3′) and 806 R (5′-GGACTACNNGGGTATCTAAT-3′). The resulting amplicons were purified using the DNA Gel/PCR Purification Small Volume Kit (Biomiga Ltd., Hangzhou, Zhejiang, China).

### 16 s rDNA sequence processing and data analysis

Purified amplicon libraries were sequenced on an Illumina MiSeq platform (Illumina, Inc., San Diego, CA, USA). Demultiplexed reads were processed with the DADA2 plugin in QIIME 2 for quality filtering, denoising, chimera removal, and inference of amplicon sequence variants (ASVs). Taxonomic classification of the resulting ASVs was conducted against the SILVA bacterial reference database. Before downstream analyzes, the ASV abundance table was rarefied to 5,625 reads per sample. Alpha-diversity metrics, including observed species, ACE, Chao1, Shannon, and Simpson indices, were calculated using the R package vegan. Microbial community variation was visualized by principal coordinate analysis based on Bray-Curtis dissimilarity, and differences between groups were evaluated using PERMANOVA. Predicted microbial metabolic pathways were inferred with PICRUSt2 and annotated according to the KEGG database.

### Fecal *in vitro* fermentation

Phosphate-buffered saline (PBS, pH 7.4) was mixed with fresh fecal samples at a ratio of 1:7 (w/v) to prepare fecal slurry for inoculation. For the gas-production validation experiment, the fecal slurry was inoculated at 10% (v/v) into two sterile anaerobic media, GMM and SM. Each fecal sample was tested in both GMM and SM, with three parallel fermentation tubes prepared for each medium condition. Fermentations were carried out in 20-mL closed anaerobic fermentation tubes sealed with rubber stoppers and incubated with shaking at 37 °C for 48 h. After fermentation, the tubes were placed on ice for 15 min to terminate microbial activity, after which the gas and fermentation broth were collected. For gas measurement, a sterile syringe fitted with a needle was inserted through the rubber stopper of each fermentation tube. Gas pressure accumulating in the headspace displaced the syringe plunger, allowing the generated gas volume to be recorded. The fermentation broth was collected for subsequent microbial and metabolite analyzes. For the *F. prausnitzii* and *B. longum* replenishment experiments, SM medium was used because it contains multiple fermentable substrates and better simulates the fermentable substrate-rich environment of colonic chyme. Fresh fecal samples from six participants with flatulence were processed as described above, and the prepared fecal slurry was inoculated into SM medium at 10% (v/v). Subsequently, *F. prausnitzii* strains A2-165 and CCFM1204, as well as 10 *B. longum* strains, were tested separately. Strain A2-165 was selected as a well-characterized reference strain of the *F. prausnitzii*, whereas CCFM1204 was selected as a healthy human feces-derived strain isolated in our laboratory. Bacterial suspensions were adjusted to 10^9^ CFU/mL before addition and were added to the fermentation system at 10% (v/v), corresponding to an initial supplemented-strain concentration of approximately 10^8^ CFU/mL in the final fermentation system.^24^ The control group received an equal volume of sterile PBS. Fermentation was performed at 37 °C with shaking for 48 h. Gas production was measured as described above, and the fermentation broth was collected. Six parallel fermentation tubes were prepared for each treatment group.

### Shotgun metagenomic sequencing and data processing

Shotgun metagenomic libraries were sequenced on a NovaSeq 6000 system (Illumina, California, USA), generating 15 Gb of sequence data. Trimmomatic v0.39 was applied to discard reads of poor quality. Human-derived reads were filtered with KneadData coupled to Bowtie2 v2.3.4.1, after which the cleaned datasets were assessed using FastQC v0.11.9. Taxonomic abundance profiles for all metagenomic samples were generated with MetaPhlAn v3.0.13. Metagenomic assembly was performed using MEGAHIT (v1.2.9), and contigs were obtained through assembly with optimized different k-mer settings. Metabat2 (v2.15) in the binning module of the metaWRAP environment was used to bin contigs and generate metagenome-assembled genomes (MAGs).^25^ CheckM (v0.4) was used to assess the quality of MAGs, and MAGs with completeness above 90% and contamination below 5% were selected.^26^ Redundancy was removed using dRep (v3.2.2), and GTDB annotation of bins was then performed using GTDB-Tk (v1.0.2). Functional annotation was performed using HUMAnN 3.0, and metabolic pathway abundance information was obtained by aligning protein sequences against the UniRef90 database. Bifidobacterium genomes were selected and annotated according to the EggNOG database to obtain COG information. Differential abundance analysis was performed on the KO abundance table generated by HUMAnN 3.0. KOs enriched in the flatulence group and healthy control group were identified using the Wilcoxon–Mann–Whitney test with Benjamini–Hochberg FDR correction, and were then separately mapped to KEGG pathways using the KEGGREST package.

### The quantification of SCFAs with GC-MS

The SCFAs concentrations were measured as described previously.^27^ Human fecal samples were pre-frozen at −80 °C and then lyophilized using a vacuum freeze dryer. Briefly, approximately 40 mg of lyophilized material was resuspended in 0.5 mL saturated NaCl and mechanically disrupted at 65 Hz for 90 s. The mixture was subsequently acidified with 40 µL H₂SO₄ and thoroughly vortexed. SCFAs were extracted by adding 1 mL of anhydrous ether, followed by centrifugation at 15,000 × g for 15 min at 4 °C. The upper organic layer was then collected and subjected to GC-MS analysis.

### H₂ and CO₂ measurement

H₂ and CO₂ concentrations in the collected fermentation gas were determined by gas chromatography using a Shimadzu GC-2010 system and an Agilent 7890 A system. H₂ was measured using a thermal conductivity detector (TCD), whereas CO₂ was measured using a flame ionization detector (FID) coupled with a methanizer. Gas samples were withdrawn from each gas collection bag using a gas-tight syringe and injected into the gas chromatographic system. H₂ and CO₂ concentrations were calculated by comparing the sample signals with those of standard gases of known concentrations.

### Untargeted metabolome by LC-MS

The metabolomics were measured as described previously.^28^ Each sample was combined with methanol at a sample-to-solvent ratio of 1:4, subjected to ultrasonic treatment for 5 min, and incubated at -20 °C for 1 h for protein precipitation. After centrifugation at 12,000 rpm and 4 °C for 15 min, the liquid fraction was collected and concentrated by centrifugal evaporation. The residue was reconstituted in chilled methanol/water solution at a volume ratio of 4:1 and centrifuged again using the same settings. The clarified extract was collected for LC–MS/MS measurement. A 2 μL aliquot was loaded onto an Agilent 1290 Infinity UPLC system and resolved using an ACQUITY UPLC BEH C18 column from Waters (1.7 μm particle size; 2.1 × 50 mm; Milford, MA, USA) at 0.35 mL/min. The aqueous eluent contained 0.1% formic acid, whereas the organic eluent consisted of acetonitrile supplemented with 0.1% formic acid. The proportion of organic eluent increased from 2% to 5% during the first minute, reached 100% over the following 11 min, remained at 100% for 3 min, and was reduced to 2% over the final 2 min. Metabolites were detected on an Agilent 6543 quadrupole time-of-flight mass spectrometer coupled to an electrospray ionization source. Full-scan spectra were recorded in positive- and negative-ion acquisition modes across an *m/z* interval of 100–1000, with a capillary potential of 3000 V.

Raw spectral files were processed in Compound Discoverer 3.1 (Thermo Fisher Scientific, MA, USA) using the standard untargeted workflow. Data processing comprised signal extraction, removal of background features, peak picking, deconvolution, chromatographic alignment, peak-area integration, and compound annotation. Metabolite assignment was performed against mzCloud, HMDB, KEGG, and ChemSpider. Features occurring in fewer than 50% of quality-control samples or showing an RSD above 30% across quality-control replicates were discarded. Mass matching was conducted with a tolerance of 5 ppm, and signals lacking a corresponding database mass match were removed. The resulting data matrix contained peak areas, retention times, molecular weights, and annotations for identified or unidentified compounds. Multivariate statistical modeling was performed in SIMCA software (Umetrics, Umeå, Sweden), and pathway-level interpretation was conducted with MetaboAnalyst 5.0.

### RT-qPCR quantification of bacteria

The absolute abundances of *B. longum* and *F. prausnitzii* in fermentation broth and fecal samples were determined by real-time quantitative PCR (RT-qPCR). total DNA was extracted from 1 mL of fermentation broth or fecal samples using the FastDNA Spin Kit according to the manufacturer’s instructions and used as the template for RT-qPCR. Each biological replicate was measured in triplicate. Standard curves were generated using the corresponding reference strains of *B. longum* and *F. prausnitzii*, and bacterial abundances were calculated based on the standard curves. The results were normalized to the volume of fermentation broth or the wet weight of fecal samples and expressed as log₁₀ CFU/mL or log₁₀ CFU/g, respectively. The primers used were as follows: primers for *B. longum* (F: 5′- TTCCAGTTGATCGCATGGTCTTCT-3′, R: 5′-GGCTACCCGTCGAAGCCACG-3′), and primers for *F. prausnitzii* (F: 5′-GGAGGAAGAAGGTCTTCGG-3′, R: 5′-AATTCCGCCTACCTCTGCACT-3′).

### Statistical analysis

In this study, unless otherwise specified, data are presented as mean ± standard error (mean ± SEM). Data processing, statistical analysis, and graphing were performed using GraphPad Prism 10, IBM SPSS Statistics, and R. In R, the vegan, picante, adonis, pheatmap, pROC and KEGGREST packages were used for data processing and analysis. Spearman's correlation coefficient was used for correlation analysis. For comparisons between two groups, the Wilcoxon-Mann-Whitney test was used for non-parametric data. For comparisons among multiple groups, the Kruskal-Wallis test was used, followed by Dunn’s multiple comparisons test with Bonferroni correction when pairwise comparisons, especially comparisons with the control group, were performed. Permutational multivariate analysis of variance (PERMANOVA) was used for multivariate non-parametric analyzes. For analysis involving multiple microbial taxa, metabolites, or multiple correlation tests, *p* values were adjusted using the Benjamini-Hochberg false discovery rate (FDR) method. In the figures, * indicates *p* < 0.05, ** indicates *p* < 0.01, *** indicates *p* < 0.001, **** indicates *p* < 0.0001, and ns indicates no significant difference (*p* > 0.05).

## Results

### Flatulence is associated with altered microbiota structure and functional potential

**Figure 1.**
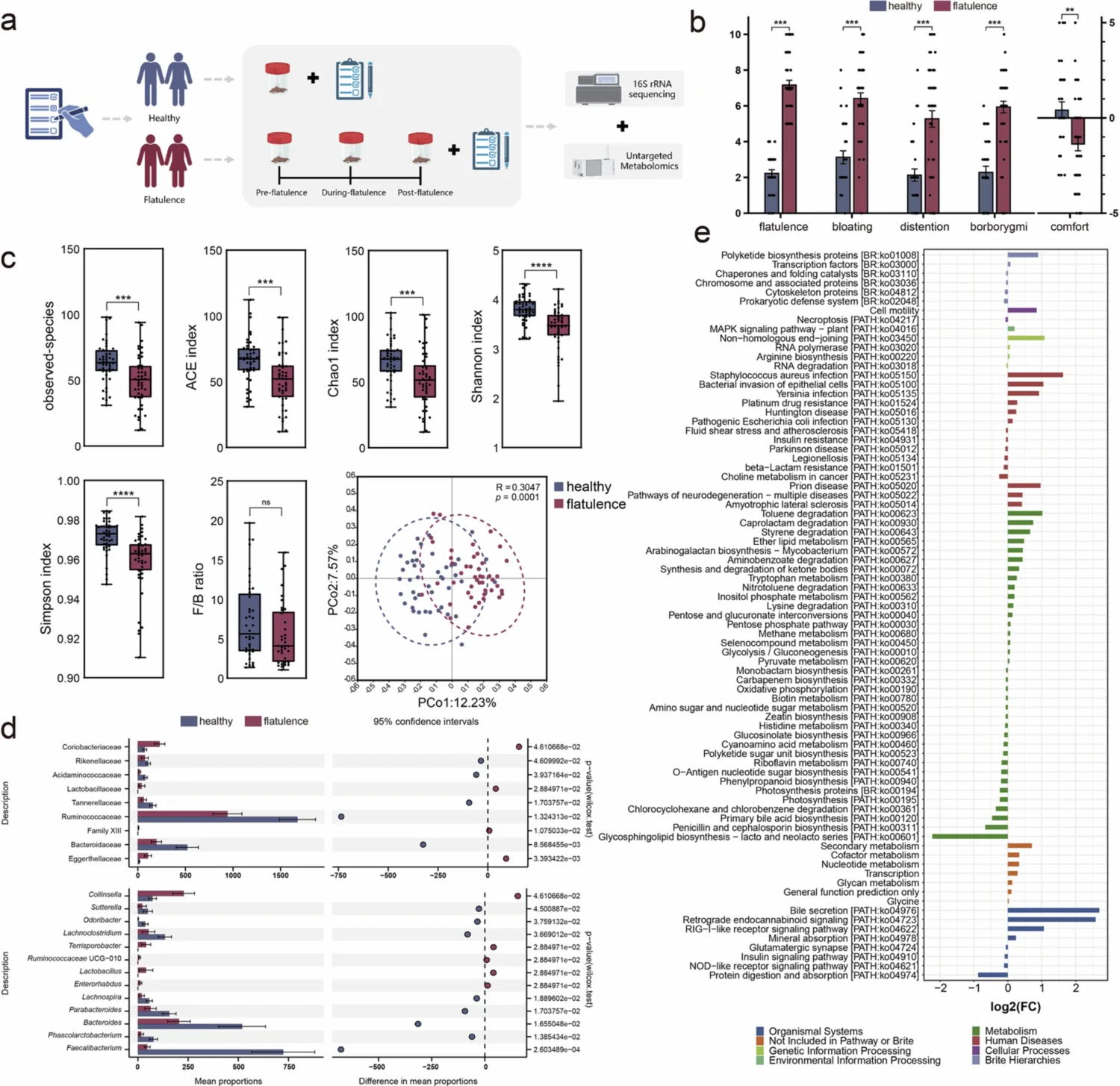
Participants with flatulence exhibit gut microbiota dysbiosis and altered predicted functional potential. (a) Schematic overview of the clinical cohort design and fecal sample collection. Participants with flatulence and healthy controls were recruited based on gastrointestinal symptom questionnaires, and fecal samples were collected for 16S rRNA gene sequencing and downstream microbial functional prediction. (b) Baseline symptom scores in healthy controls and participants with flatulence, including flatulence, bloating, abdominal distension, borborygmi, and gastrointestinal comfort. (c) Comparison of gut microbial *α*-diversity and *β*-diversity between healthy controls and participants with flatulence. *α*-diversity indices included observed species, ACE, Chao1, Shannon, and Simpson indices; the Firmicutes/Bacteroidetes ratio was also compared. *β*-diversity was visualized by principal coordinate analysis based on Bray-Curtis dissimilarity. (d) Differential microbial taxa between the two groups identified using the Wilcoxon rank-sum test and STAMP analysis. (e) Differences in predicted gut microbial functional potential inferred by PICRUSt2. Bars indicate the log₂ fold change of differential KEGG pathways and are colored according to KEGG functional categories. Positive values indicate enrichment in participants with flatulence, whereas negative values indicate enrichment in healthy controls.

A total of 76 participants were recruited based on symptom questionnaires, including 44 participants with self-reported flatulence and 32 age- and sex-matched healthy controls. The mean age of the cohort was 24 years, the mean BMI was 21.67 ± 2.54, and 69.7% of the participants were female (Table S1). This sex distribution is broadly consistent with previous epidemiological reports showing that women are three to four times more likely than men to report flatulence.^29^ ^,^ ^30^ The baseline symptom scores of participants in the flatulence and healthy control groups included flatulence, bloating, abdominal distension, borborygmi, and digestive tract comfort (Figure 1b). The flatulence scores in the flatulence and healthy control groups were 7.159 ± 0.272 and 2.219 ± 0.209, respectively. To identify potential microbial mechanisms associated with flatulence, fecal samples from both groups were subjected to 16S rRNA gene sequencing. Compared with healthy controls, participants with flatulence showed marked gut microbial dysbiosis, characterized by a significant reduction in the number of annotated species, decreased richness indices and diversity indices. The Firmicutes/Bacteroidetes ratio did not differ significantly between the two groups. PCoA based on Bray-Curtis dissimilarity and PERMANOVA revealed a significant difference in microbial community composition between participants with flatulence and healthy controls (*R* = 0.3047, *p* = 0.0001) (Figure 1c). In addition, Adonis multivariate analysis showed that symptom status explained more inter-sample variation than sampling time (symptom status: *R^2^* = 0.0270, *p* < 0.0001; sampling time: *R^2^* = 0.0086, *p* > 0.05; Figure S3a), suggesting that flatulence-associated microbial features were relatively stable across sampling time points. Therefore, the second fecal samples (collected during-flatulence) from participants with flatulence were used as representative samples for subsequent analyzes.

At the taxonomic level, participants with flatulence showed an increased abundance of Actinobacteria and a decreased abundance of Bacteroidetes. At the family level, Coriobacteriaceae, Lactobacillaceae, and Eggerthellaceae were significantly enriched, whereas Ruminococcaceae and Bacteroidaceae were significantly reduced. At the genus level, 13 genera differed significantly between the two groups. Among them, *Collinsella*, *Terrisporobacter*, *Ruminococcaceae UCG-010*, *Lactobacillus*, and *Enterorhabdus* were significantly increased in the flatulence group. In contrast, *Parabacteroides*, *Bacteroides*, and *Faecalibacterium* were significantly decreased (Figure 1d and Figure S3b-d). We further predicted the functional potential of the gut microbiota using PICRUSt2. Multiple pathways related to carbohydrate utilization and energy metabolism were significantly enriched in the flatulence group, including pyruvate metabolism, the pentose phosphate pathway, methane metabolism, glycolysis/gluconeogenesis, and glycan metabolism. In addition, several KEGG pathways annotated as bacterial infection-related pathways, such as *Staphylococcus aureus* infection, Yersinia infection, and pathogenic *Escherichia coli* infection, also showed an increasing trend in participants with flatulence (Figure 1e). Together, these results suggest that participants with flatulence exhibit not only reduced microbial diversity and altered community structure, but also changes in potential carbohydrate fermentation capacity and disease-related functional modules.

### Flatulence associated microbiota show enhanced gas production linked to reduced *F. prausnitzii*

**Figure 2.**
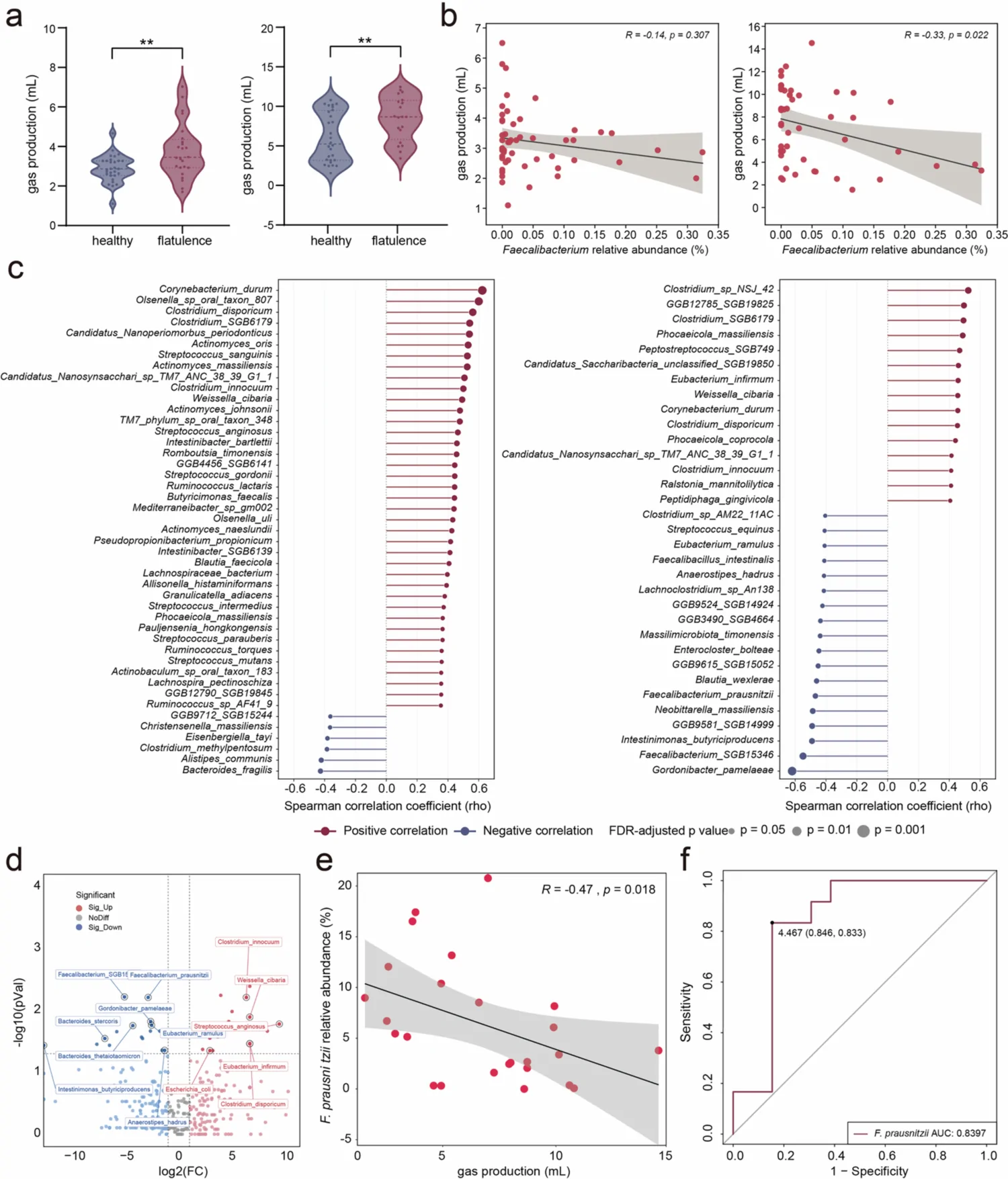
Flatulence associated microbiota shows enhanced gas-producing capacity linked to reduced *F. prausnitzii.* (a) Comparison of *in vitro* gas production by fecal microbiota from healthy controls and participants with flatulence under different culture medium conditions. The left panel shows total gas production under GMM conditions, and the right panel shows total gas production under SM conditions. (b) Correlations between genus-level *Faecalibacterium* relative abundance and in vitro gas production. The left panel shows fermentation under GMM conditions, and the right panel shows fermentation under SM conditions. Correlations were assessed using Spearman correlation analysis. (c) Lollipop plots showing species significantly associated with gas production. The x-axis represents Spearman’s correlation coefficient (rho), and each point represents one species. The left panel shows fermentation under GMM conditions, and the right panel shows fermentation under SM conditions. Red indicates a positive correlation with gas production, whereas blue indicates a negative correlation. Point size represents the FDR-adjusted *p* value, with larger points indicating smaller *p* values. (d) Volcano plot showing differentially enriched species between the high- and low-gas-producing groups. (e) Correlation between the relative abundance of *F. prausnitzii* and *in vitro* gas production. Correlation was assessed using Spearman correlation analysis. (f) Receiver operating characteristic curve analysis evaluating the ability of *F. prausnitzii* relative abundance to distinguish high- and low-gas-producing phenotypes. The area under the curve indicates the discriminatory performance of the model.

After identifying alterations in the gut microbiota structure of participants with flatulence, we further performed *in vitro* fermentation using fresh fecal samples to evaluate the gas-producing capacity of microbiota from different groups under the same substrate conditions. Two media were used: GMM containing basal substrates and SM, a simulated colonic chyme medium enriched in fermentable substrates. The results showed that, under both medium conditions, fecal microbiota from participants with flatulence produced significantly more gas than that from healthy controls (GMM: *p* = 0.0081; SM: *p* = 0.0093) (Figure 2a). In addition, compared with GMM, the fermentable substrate-rich SM medium induced higher gas production, consistent with the expectation that fermentable substrates promote microbial gas generation. We then ranked the fermentation samples according to gas production and analyzed the corresponding genus-level microbial composition. As gas production increased, the genus-level relative abundance of *Faecalibacterium* markedly decreased in high-gas-producing samples (Figure S4a). Moreover, *Faecalibacterium* was significantly negatively correlated with gas production under the SM condition (Figure 2b). These findings suggest that specific microbial community structures may be closely associated with the gas-producing capacity of the gut microbiota.

To further resolve gas-associated taxa at the species level, shotgun metagenomic sequencing was performed on fecal samples. A total of 995 species were annotated. After quality control filtering to remove species with a prevalence below 10% or relative abundance below 0.01%, 443 species were retained for correlation analysis. We analyzed the correlations between species relative abundance and gas production separately in the GMM and SM fermentation conditions and further constructed correlation networks among gas-associated species. In the GMM medium, 45 species were significantly associated with gas production (*p* < 0.05), including 39 positively correlated and 6 negatively correlated species. The positively correlated species mainly included members of Lachnospiraceae, such as *Lachnospira pectinoschiza* and *Lachnospiraceae bacterium*; members of *Ruminococcus*, including *Ruminococcus torques* and *Ruminococcus lactaris*; members of *Streptococcus*, including *Streptococcus mutans*, *Streptococcus anginosus*, *Streptococcus sanguinis*, and *Streptococcus gordonii*; and members of *Clostridium*, including *Clostridium innocuum*, *Clostridium SGB6179*, and *Clostridium disporicum*. In contrast, *Bacteroides fragilis*, *Alistipes communis*, and *GGB9712_SGB15244* were significantly negatively correlated with gas production (Figure 2c and Figure S4b). In the SM medium, 33 species were significantly associated with gas production (*p* < 0.05), including 15 positively correlated and 18 negatively correlated species. Similar to the results in the GMM system, several *Clostridium* members, including *Clostridium innocuum*, *Clostridium disporicum*, *Clostridium SGB6179*, and *Clostridium sp. NSJ-42*, together with *Peptostreptococcus SGB749*, were positively correlated with gas production. Notably, *Faecalibacterium SGB15346* and *Faecalibacterium prausnitzii* were significantly negatively correlated with gas production in the SM system, suggesting that *Faecalibacterium*-related species may be associated with a low-gas-producing phenotype (Figure 2c and Figure S4b). To further focus on the key species most closely associated with the gas-production phenotype, we amplified the contrast in gas production by comparing the top 10 highest-gas-producing and top 10 lowest-gas-producing samples. *F. prausnitzii* was significantly enriched in the low-gas group, and correlation analysis showed that the relative abundance of *F. prausnitzii* was significantly negatively correlated with gas production (*R* = −0.47, *p* = 0.018) (Figure 2d−e). In addition, receiver operating characteristic curve analysis based on the relative abundance of *F. prausnitzii* yielded an AUC of 0.8397 (Figure 2f), suggesting that *F. prausnitzii* has potential as a candidate biomarker associated with the low-gas-producing phenotype.

### Participants with flatulence exhibit oxidative stress-related functional dysbiosis and fecal inflammatory signatures

**Figure 3.**
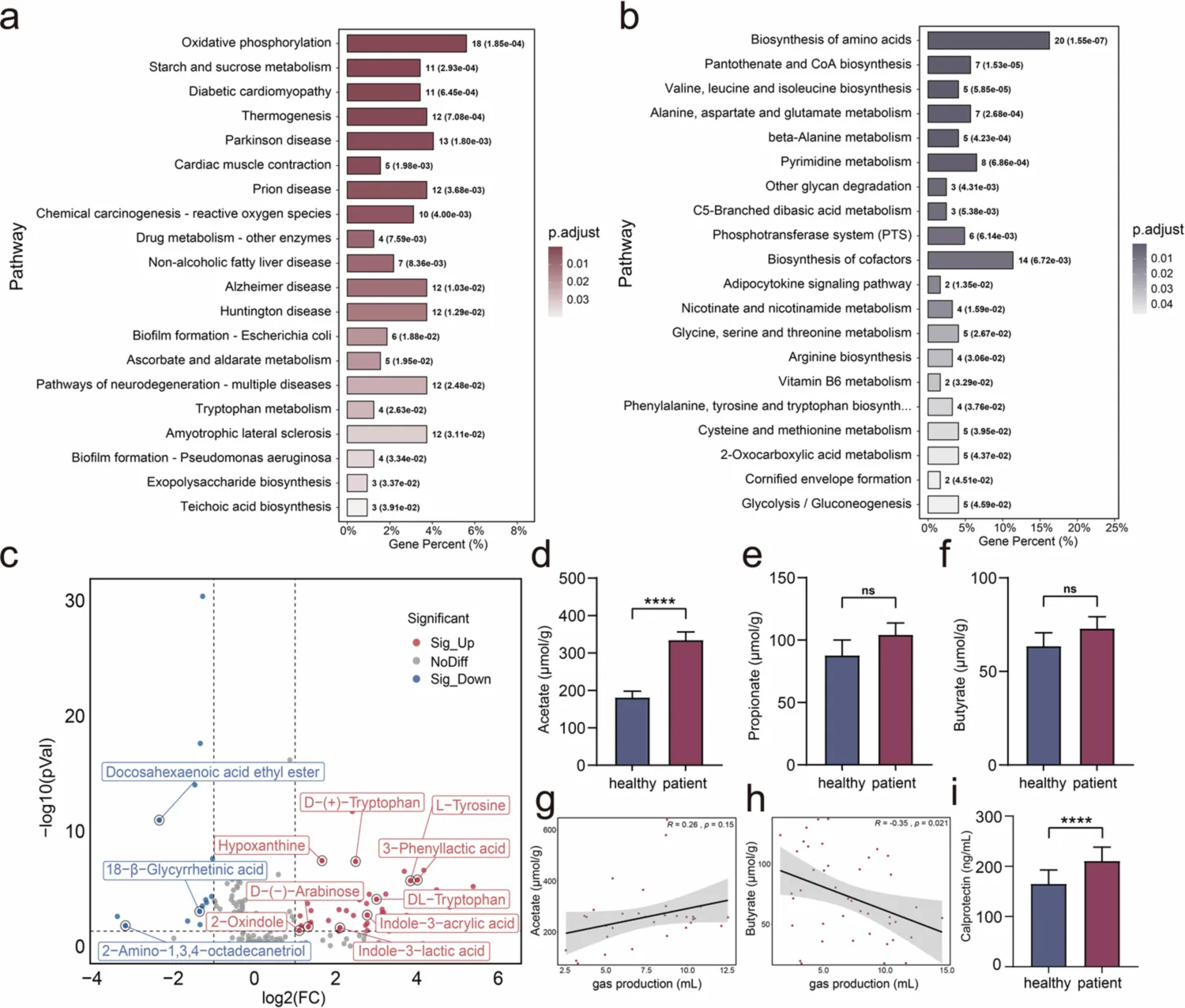
Fecal microbial functional dysbiosis, metabolic alterations, and inflammatory signatures in participants with flatulence. (a−b) KEGG pathway enrichment results based on differentially abundant KOs enriched in the flatulence group (a) and healthy control group (b). Bars indicate the gene percentage of significantly enriched pathways, and colors represent the adjusted significance level. (c) Volcano plot showing differential fecal metabolites between healthy controls and participants with flatulence. Red dots indicate metabolites significantly increased in the flatulence group, blue dots indicate metabolites significantly decreased in the flatulence group, and gray dots indicate metabolites without significant differences. Dashed lines indicate the fold-change thresholds. (d-f) Comparison of fecal short-chain fatty acid levels between healthy controls and participants with flatulence, including acetate (d), propionate (e), and butyrate (f). (g-h) Correlation analysis between fecal acetate (g) or butyrate (h) levels and *in vitro* gas production. The solid line indicates the fitted linear regression curve, and the shaded area represents the 95% confidence interval. (i) Comparison of fecal calprotectin concentrations between healthy controls and participants with flatulence.

Along with alterations in gut microbial composition and gas-production characteristics, the metagenomic functional profile of participants with flatulence was also markedly remodeled. KEGG pathway enrichment analysis based on microbial functional annotation showed that oxidative phosphorylation (ko00190), Chemical carcinogenesis-reactive oxygen species (ko05208), Biofilm formation-*Escherichia coli* (ko02026), and Biofilm formation-*Pseudomonas aeruginosa* (ko02025) were significantly enriched in the flatulence group. In addition, pathways related to carbohydrate fermentation, such as Starch and sucrose metabolism, as well as amino acid biosynthetic networks, also showed substantial metabolic remodeling (Figure 3a). In contrast, microbial functions enriched in the healthy group were mainly associated with Biosynthesis of amino acid, Biosynthesis of cofactors, and substrate utilization, including valine, leucine, and isoleucine biosynthesis; alanine, aspartate, and glutamate metabolism; glycine, serine, and threonine metabolism; arginine biosynthesis; phenylalanine, tyrosine, and tryptophan biosynthesis; and cysteine and methionine metabolism. In addition, pathways involved in Pantothenate and CoA biosynthesis, nicotinate and nicotinamide metabolism, and vitamin B6 metabolism were also enriched in the healthy control group. To evaluate the metabolic outputs associated with these microbial functional alterations, untargeted LC-MS-based metabolomics was performed on fecal samples. Among the 183 quantified metabolites, 56 metabolites differed significantly between the two groups. Of these, 42 metabolites were significantly enriched in the flatulence group, including aromatic amino acids and putrefactive fermentation products, such as DL-Tryptophan, L-Tyrosine, and several indole derivatives. Notably, Hypoxanthine, a marker associated with oxidative stress, and the poorly absorbed sugar D-(-)-Arabinose were also significantly enriched in the flatulence group. In contrast, lipid metabolites with potential anti-inflammatory and mucosal protective properties, including docosahexaenoic acid ethyl ester, 18-*β*-Glycyrrhetinic acid, and Phytosphingosine, were significantly depleted in the flatulence group (Figure 3c).

Because gas production by the gut microbiota is closely linked to the production and consumption of SCFAs, fecal SCFAs were quantified and correlated with gas production. SCFAs analysis showed that fecal acetate levels were markedly increased in the flatulence group (*p* < 0.0001; Figure 3d), and acetate levels were positively correlated with *in vitro* gas production in the simulated colonic chyme medium (Figure 3g). Although propionate (Figure 3e) and butyrate (Figure 3f) levels did not differ significantly between groups, butyrate levels were significantly negatively correlated with gas production (Figure 3h), suggesting that higher gas production may be associated with reduced butyrate synthesis. Finally, fecal calprotectin was measured to assess host inflammatory signatures. Fecal calprotectin concentrations were significantly higher in the flatulence group than in healthy controls (*p* < 0.0001; Figure 3i).

### *F. prausnitzii* replenishment reduces gas production and promotes butyrate-associated microbiota remodeling

**Figure 4.**
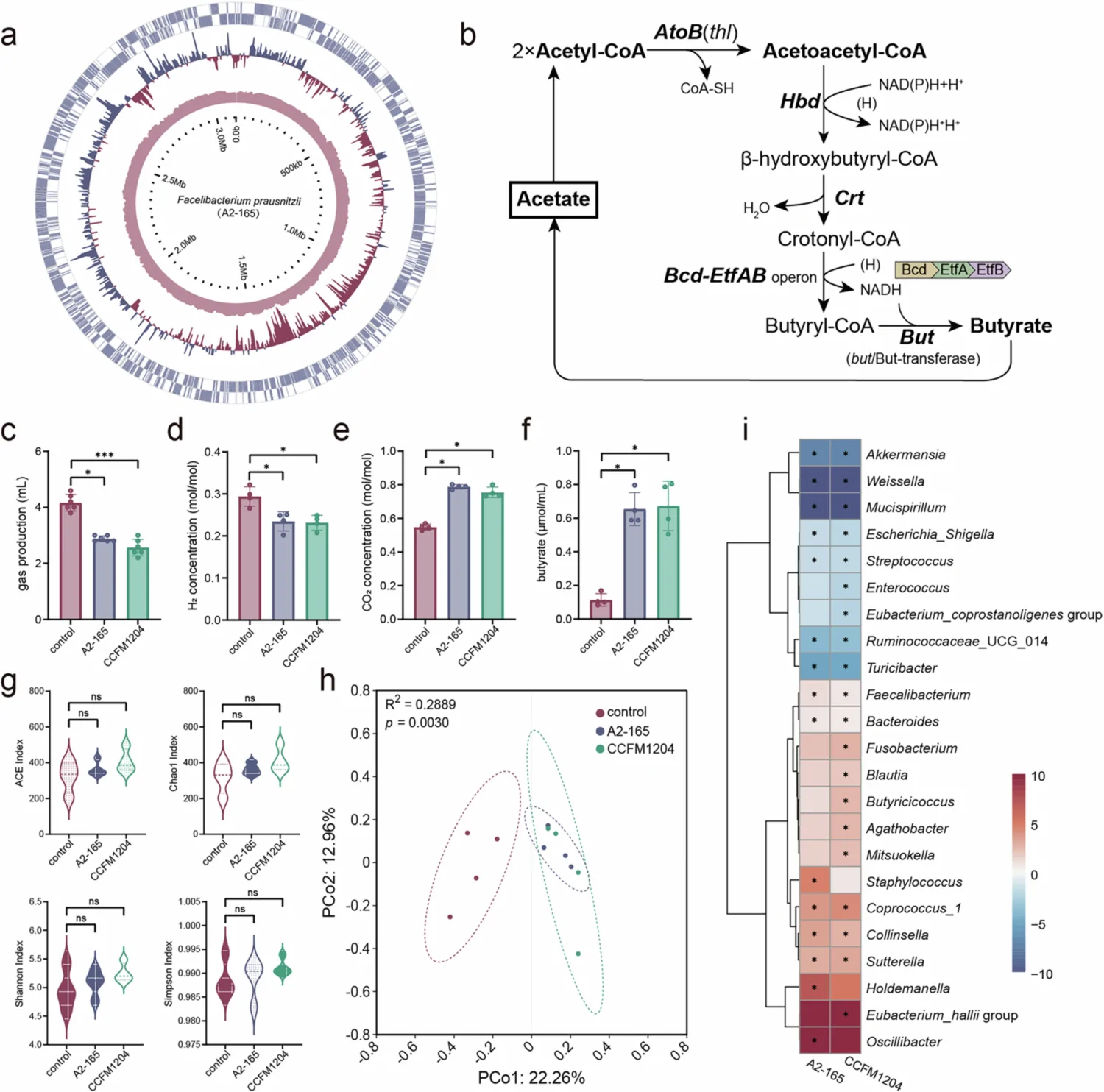
*F. prausnitzii* replenishment reduces gas production and promotes butyrate-associated microbiota remodeling. (a) Circular genome map of *F. prausnitzii* A2-165 showing the overall genomic architecture, including coding sequences, GC content, and GC skew distribution. (b) Schematic representation of the acetate-dependent butyrate biosynthesis pathway in *F. prausnitzii*. (c-f) Effects of *F. prausnitzii* A2-165 and CCFM1204 replenishment on the *in vitro* fermentation phenotype of flatulence-associated microbiota, including total gas production (c), H₂ concentration (d), CO₂ concentration (e), and butyrate concentration (f). (g) Changes in microbial *α*-diversity after *F. prausnitzii* replenishment, including ACE, Chao1, Shannon, and Simpson indices. (h) PCoA based on Bray–Curtis dissimilarity showing changes in microbial *β*-diversity of the fermentation system after *F. prausnitzii* replenishment. Ellipses indicate 95% confidence intervals for each treatment group. (i) Microbial compositional changes in the fermentation system after *F. prausnitzii* replenishment. The heatmap shows the log₂ fold change in genus-level relative abundance in the A2-165 and CCFM1204 groups compared with the non-replenished control group. Asterisks indicate FDR *p* < 0.05.

Based on the observed associations of *F. prausnitzii* with the low-gas-producing phenotype and butyrate levels, we further selected the reference strain *F. prausnitzii* A2-165 and the healthy human feces-derived strain CCFM1204 for *in vitro* replenishment fermentation to evaluate their effects on gas-production characteristics and metabolic outputs of flatulence-associated microbiota. First, we performed a genome overview and functional annotation of *F. prausnitzii* A2-165. The circular genome map showed the overall genomic features of A2-165, including coding sequences, GC content, and GC skew distribution (Figure 4a). Based on the functional annotation, we further analyzed its metabolic potential for butyrate synthesis. Pathway annotation showed that *F. prausnitzii* mainly produces butyrate through an acetate-dependent butyrate synthesis pathway. In this pathway, two molecules of acetyl-CoA serve as the initial substrates and are converted to acetoacetyl-CoA by acetyl-CoA acetyltransferase *AtoB/Thl*. Acetoacetyl-CoA is subsequently converted to *β*-hydroxybutyryl-CoA by 3-hydroxybutyryl-CoA dehydrogenase *Hbd*, and then to crotonyl-CoA by enoyl-CoA hydratase *Crt*. Crotonyl-CoA is further converted to butyryl-CoA through the butyryl-CoA dehydrogenase complex *Bcd-EtfAB*. Finally, butyryl-CoA reacts with exogenous acetate via butyryl-CoA:acetate CoA-transferase *But*, resulting in CoA transfer and butyrate production (Figure 4b). This pathway indicates that butyrate production by *F. prausnitzii* is closely linked to acetate utilization. In the *in vitro* fermentation system, compared with the non-replenished control group, replenishment with either *F. prausnitzii* A2-165 or CCFM1204 significantly reduced total gas production, with CCFM1204 showing a stronger effect than the reference strain (Figure 4c). We further analyzed the composition of the collected gas to determine how *F. prausnitzii* replenishment affected fermentation gas profiles. Compared with the non-replenished control group, in which no additional *F. prausnitzii* strain was added, both *F. prausnitzii* strains significantly reduced H₂ concentration (Figure 4d), whereas CO₂ concentration was relatively increased (Figure 4e), suggesting that *F. prausnitzii* replenishment not only reduced total gas production but also altered gas composition. Meanwhile, SCFAs analysis showed that both A2-165 and CCFM1204 significantly increased butyrate levels in the fermentation system (Figure 4f). This result is consistent with the annotated complete acetate-dependent butyrate synthesis pathway and indicates that *F. prausnitzii* replenishment can enhance butyrate production *in vitro*.

We next assessed the effect of *F. prausnitzii* replenishment on microbial diversity after fermentation. *α*-diversity analysis showed that ACE, Chao1, Shannon, and Simpson indices did not differ significantly between the A2-165 or CCFM1204 replenishment groups and the control group (Figure 4g), indicating that *F. prausnitzii* replenishment did not markedly alter the overall richness or diversity of the fermentation microbiota. In contrast, PCoA showed clear separation among treatment groups, and PERMANOVA analysis indicated a statistically significant difference in community structure (*R^2^* = 0.2889, *p* = 0.0030) (Figure 4h). These results suggest that *F. prausnitzii* replenishment mainly reshaped microbial community composition rather than broadly altering overall diversity. We then analyzed the microbial composition after *F. prausnitzii* replenishment. Compared with the control group, both A2-165 and CCFM1204 significantly decreased the relative abundance of *Weissella*, *Escherichia-Shigella*, *Streptococcus*, and *Ruminococcaceae UCG-014*, genera that were positively associated with the high-gas-producing phenotype in the previous analysis. At the same time, *F. prausnitzii* replenishment significantly increased the abundance of several strictly anaerobic butyrate-producing taxa, including *Butyricicoccus*, *Agathobacter*, *Coprococcus*, and the *Eubacterium hallii* group (Figure 4i). These findings suggest that *F. prausnitzii* replenishment remodeled the composition of flatulence-associated microbiota and promoted a shift toward a low-gas-producing and high-butyrate-producing community state. Finally, we characterized metabolite changes after *F. prausnitzii* replenishment in the *in vitro* fermentation system. Replenishment with A2-165 and CCFM1204 significantly increased 2-hydroxycaproic acid, 2-hydroxy-4-methylthiobutanoic acid, 5-hydroxytryptophan, indole-3-lactic acid, and 3-hydroxybutyric acid. In addition, CCFM1204 significantly decreased 2-hydroxyphenylalanine, DL-4-hydroxyphenyllactic acid, and cholic acid. Collectively, these results demonstrate that *F. prausnitzii* replenishment reduces the *in vitro* gas-producing capacity of flatulence-associated microbiota, decreases H₂ accumulation, enhances butyrate production, and reshapes microbial composition and metabolic outputs (Figure S5).

### *B. longum* CCFM1319 enriches *F. prausnitzii* through a cross-feeding-based strategy

**Figure 5.**
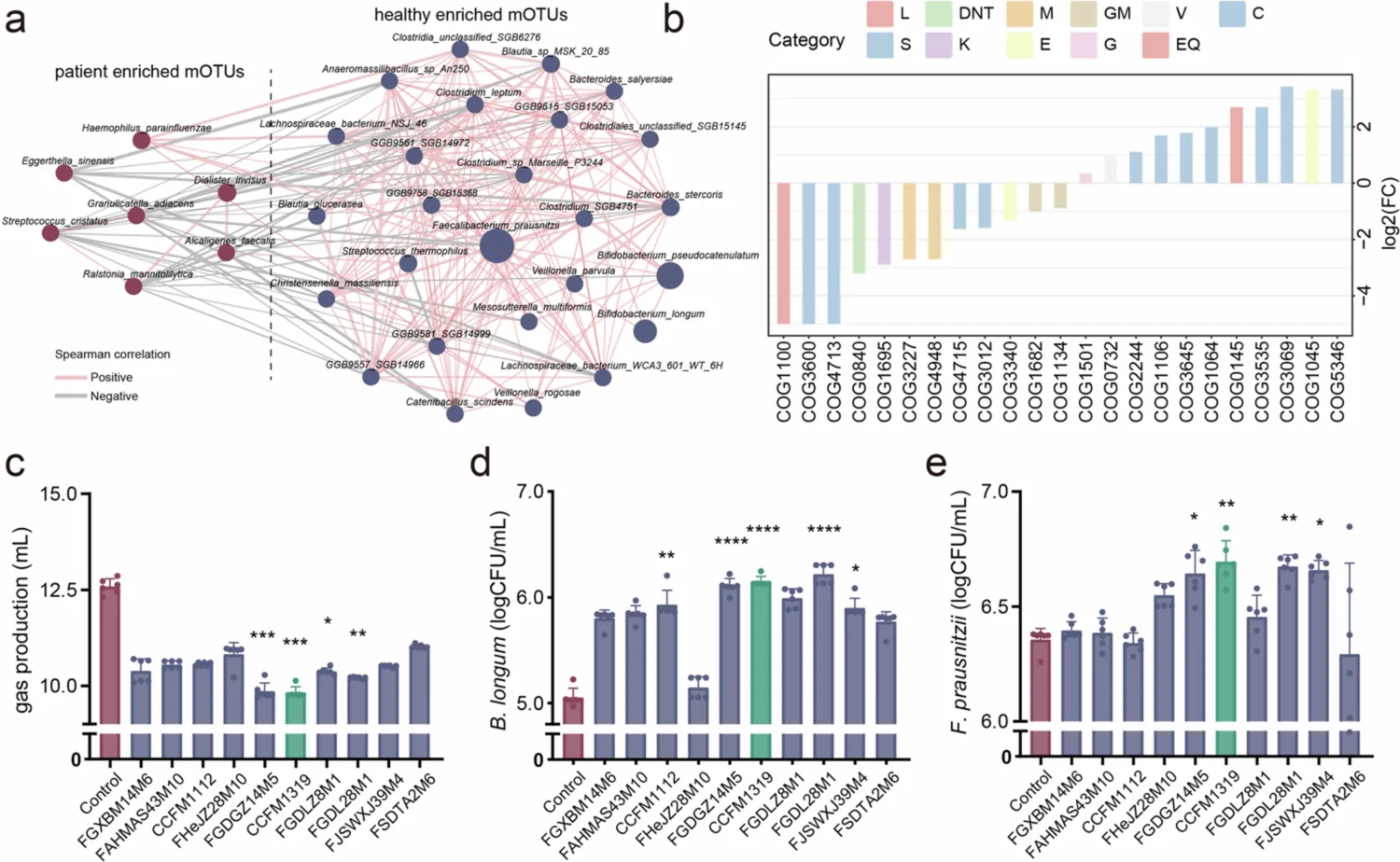
A cross-feeding-based screening strategy identifies *B. longum* CCFM1319 as a candidate strain for enriching *F. prausnitzii.* (a) Correlation network of differentially enriched species between participants with flatulence and healthy controls. Nodes represent differentially enriched species, and edges indicate interspecies correlations; red edges indicate positive correlations, whereas gray edges indicate negative correlations. Node colors distinguish species enriched in participants with flatulence from those enriched in healthy controls, and node size represents relative abundance. (b) COG difference analysis of *Bifidobacterium* between participants with flatulence and healthy controls. Bars indicate the relative changes in differential COGs, and colors represent COG functional categories. (c-e) Effects of supplementation with different *B. longum* strains on fermentation phenotypes and key bacterial abundances in flatulence-associated microbiota, including total gas production (c), absolute abundance of *B. longum* in the fermentation broth (d), and absolute abundance of *F. prausnitzii* (e). Red indicates the blank control without *B. longum* supplementation, green indicates CCFM1319, and blue indicates the other *B. longum* strain groups.

Based on the above *in vitro* validation results, we identified *F. prausnitzii* as a potential intervention target for alleviating flatulence. Although the potential of *F. prausnitzii* as a next-generation probiotic has been widely reported, it is not currently included in the list of edible bacterial strains. Therefore, enriching *F. prausnitzii* through microbial cross-feeding may represent a more feasible strategy. To screen potential targeting strains, we constructed a correlation network of differentially enriched species between participants with flatulence and healthy controls, aiming to identify species potentially associated with *F. prausnitzii* that could serve as cross-feeding partners and regulate its abundance through microbial interactions. The results showed that, in addition to the significant enrichment of *F. prausnitzii* in healthy controls, *Bifidobacterium longum* and *Bifidobacterium pseudocatenulatum* were also significantly enriched and positively correlated with the abundance of *F. prausnitzii*. We then assembled the gene sequences from both groups and further annotated the assembled *Bifidobacterium* genomes against the EggNOG database to obtain COG functional profiles for differential analysis. The results showed clear differences in *Bifidobacterium*-associated functions between healthy controls and participants with flatulence, mainly involving amino acid transport and metabolism (E), cell wall/membrane/envelope biogenesis (M), and carbohydrate transport and metabolism (G). Among these functions, GH30 family protein (COG3227), GH31 family protein (COG1501), ABC transporter ATP-binding protein (COG1134), and ABC transporter permease protein (COG1682) were significantly reduced in participants with flatulence. These results indicate functional differences in *Bifidobacterium* between the two groups, suggesting that the capacity for carbohydrate uptake and degradation by intestinal *Bifidobacterium* may be reduced in participants with flatulence, which may limit its growth advantage in the gut. Based on these findings, we selected 10 *B. longum* strains for *in vitro* fermentation screening to evaluate their effects on gas production by flatulence-associated microbiota, *F. prausnitzii* abundance, and their own ability to gain a growth advantage. Compared with the non-supplemented control group, four *B. longum* strains significantly reduced gas production, namely FGDGZ14M5, CCFM1319, FGDLZ8M1, and FGDL28M1, among which FGDGZ14M5 and CCFM1319 showed the most pronounced effects (*p* < 0.001) (Figure 5c). Meanwhile, the viable counts of *B. longum* CCFM1112, FGDGZ14M5, CCFM1319, FGDLZ8M1, FGDL28M1, and FJSWXJ39M4 were significantly increased, indicating that these strains could better gain a growth advantage in the fermentation system (Figure 5d). Further quantification of the absolute abundance of *F. prausnitzii* showed strain-specific effects of different *B. longum* strains on *F. prausnitzii* enrichment, with CCFM1319 showing the strongest promoting effect (Figure 5e). Considering both gas suppression and *F. prausnitzii* enrichment capacity, *B. longum* CCFM1319 was selected for subsequent human intervention validation.

### *B. longum* CCFM1319 intervention improves flatulence-related symptoms and increases fecal *F. prausnitzii* abundance

**Figure 6.**
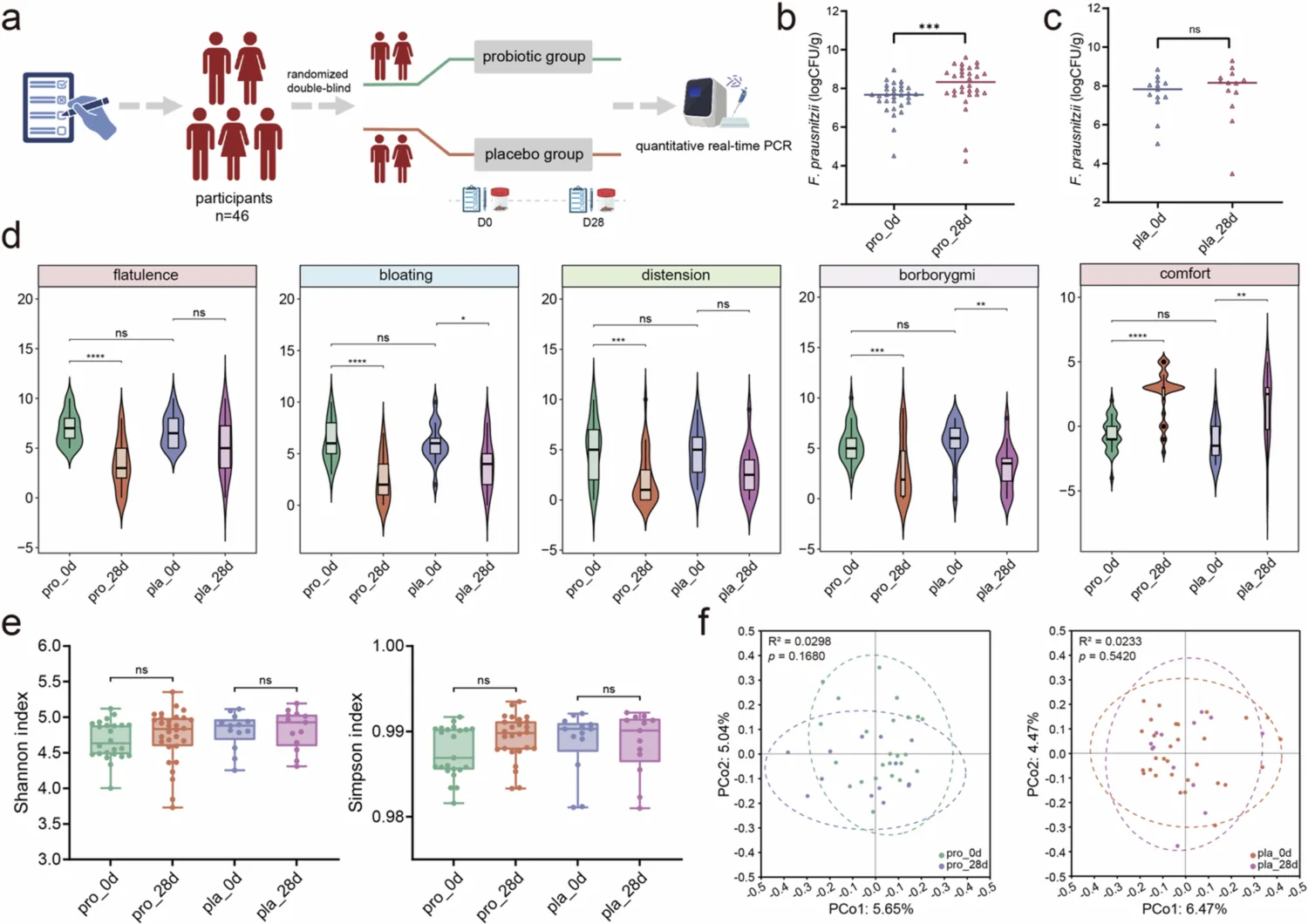
*B. longum* CCFM1319 intervention improves flatulence-related symptoms and increases fecal *F. prausnitzii* abundance. (a) Schematic overview of the clinical intervention with *B. longum* CCFM1319. (b-c) Changes in fecal *F. prausnitzii* absolute abundance before and after intervention in the CCFM1319 group (b) and placebo group (c). (d) Changes in flatulence-related clinical symptom scores before and after CCFM1319 intervention, including flatulence, bloating, abdominal distension, borborygmi, and digestive tract comfort. (e) Comparison of gut microbial *α*-diversity before and after intervention in the CCFM1319 and placebo groups, including the Shannon index and Simpson index. (f) Principal coordinate analysis based on Bray–Curtis dissimilarity of *β*-diversity before and after intervention in the CCFM1319 and placebo groups. Ellipses indicate 95% confidence intervals.

We then conducted a clinical intervention trial using *B. longum* CCFM1319 to validate its effect in participants with flatulence. Participants with flatulence were screened based on flatulence symptom questionnaires, and 46 eligible participants were enrolled; their baseline characteristics are shown in Table S2. Participants were randomly assigned to the CCFM1319 group or placebo group at a 2:1 ratio, allowing us to obtain more data on symptom responses, gut microbiota changes, and safety after CCFM1319 intervention while maintaining a placebo-controlled comparison. Fecal samples and clinical symptom scores were collected before and after the 28-day intervention (Figure 6a). Baseline flatulence symptom scores did not differ significantly between the two groups (Figure S7b). During the intervention period, no adverse events related to sample intake were reported in either group, indicating good clinical tolerability of CCFM1319. The GSRS results showed that the total score in the placebo group changed from 38.18 ± 12.07 to 34.33 ± 11.00, without a significant difference, whereas the score in the CCFM1319 group decreased significantly from 40.83 ± 11.90 to 30.24 ± 11.51, suggesting improvement in overall gastrointestinal discomfort (Figure S7c). We further evaluated flatulence-related symptoms. After 28 days of intervention, all five symptom scores were significantly improved in the CCFM1319 group: the flatulence score decreased from 6.933 ± 0.295 to 3.333 ± 0.448, the bloating/pressure score decreased from 6.133 ± 0.361 to 2.567 ± 0.400, the abdominal distension score decreased from 4.933 ± 0.549 to 2.100 ± 0.463, the borborygmi score decreased from 5.433 ± 0.328 to 3.000 ± 0.538, and the gastrointestinal comfort score increased from −0.600 ± 0.233 to 2.533 ± 0.342 (Figure 6d). In contrast, the placebo group showed improvement only in bloating/pressure, borborygmi, and gastrointestinal comfort, whereas flatulence and abdominal distension scores did not change significantly (Figure 6d). We then assessed the effect of CCFM1319 intervention on overall gut microbial diversity. The results showed that neither the CCFM1319 group nor the placebo group exhibited significant changes in *α*-diversity before and after intervention, although the Simpson index in the CCFM1319 group showed an increasing trend without reaching statistical significance (*p* = 0.0557) (Figure 6e). PCoA analysis also showed no significant differences in overall gut microbial structure between the two groups at baseline or at the end of intervention (D0: *p* = 0.1680; D28: *p* = 0.5420) (Figure 6f). Finally, we performed absolute quantification of fecal *F. prausnitzii*. After 28 days of CCFM1319 intervention, fecal *F. prausnitzii* abundance increased significantly from 9.43 × 10^7^ CFU/g to 6.25 × 10^8^ CFU/g, corresponding to an approximately 6.63-fold increase (Figure 6b). In contrast, no significant change in *F. prausnitzii* abundance was observed in the placebo group before and after intervention (Figure 6c). These results indicate that CCFM1319 intervention can increase the absolute abundance of intestinal *F. prausnitzii* and improve flatulence-related symptoms.

## Discussion

In this study, we integrated a clinical cohort, *in vitro* fermentation, metagenomic analysis, and metabolomics to systematically characterize the gut microbiota and metabolic features of participants with flatulence. We further investigated the potential role of *F. prausnitzii* as a microbial target and conducted functional validation together with a clinical intervention using *B. longum* CCFM1319. Gut microbial diversity is widely regarded as an important indicator of microbial ecological homeostasis. Previous studies have shown that reduced microbial diversity is commonly observed in gastrointestinal diseases or disorders, including IBS, IBD, and recurrent *Clostridioides difficile*-associated diarrhea,^31-34^ whereas higher species richness is generally associated with greater ecological redundancy, stronger resilience, and increased resistance to colonization by potential pathogens.^35^ In the present study, participants with flatulence showed significantly lower microbial diversity than healthy controls, consistent with the findings of Ringel-Kulka et al.^7^ At the compositional level, participants with flatulence were characterized by enrichment of multiple potentially fermentative taxa together with depletion of strict anaerobic commensals. Eggerthellaceae was enriched in the gut microbiota of participants with flatulence, and this bacterial family has previously been associated with depression,^36^ which may be related to increased psychological stress in some participants with flatulence. Meanwhile, *Parabacteroides*, *Bacteroides*, and *Faecalibacterium* were significantly reduced, which may weaken intestinal anaerobic homeostasis and protective microbial networks, thereby creating ecological space for the expansion of facultative anaerobic and less fastidious bacteria. Notably, although *Lactobacillus* is generally considered to have probiotic potential, its increase under certain intestinal ecological conditions has also been associated with abdominal pain and bloating in patients with IBS. Lactobacilli can utilize glucose or fructose to produce lactate and acetate, accompanied by fermentative products such as CO₂. Similarly, *Collinsella* can degrade disaccharides and oligosaccharides to produce acetate, lactate, formate, and H₂,^37^ and may therefore contribute to acetate accumulation and enhanced gas generation in participants with flatulence. Species-level associations with gas production further showed that Lachnospiraceae, *Ruminococcus*, *Streptococcus*, and *Clostridium* were positively correlated with gas production. Members of Lachnospiraceae and *Ruminococcus* are capable of degrading complex carbohydrates, including pectin, polygalacturonic acid, fructose, and cellobiose, producing acetate, formate, ethanol, CO₂, and small amounts of H₂.^38^ *Clostridium* has also been closely associated with intestinal H₂ production. Manichanh et al. reported that participants with flatulence had increased gas evacuation, particularly after consuming more fermentable diets.^2^ James et al. also reported that Firmicutes represent one of the major sources of intestinal hydrogen production and are relatively increased in patients with IBS, whereas Bacteroidetes are reduced, a pattern that may be related to bloating symptoms.^39^ In contrast, certain non-enterotoxigenic *Bacteroides fragilis* strains have been suggested to strengthen the intestinal epithelial barrier and inhibit pathogen colonization. In the present study, *B. fragilis* was negatively correlated with the gas-associated species *Lachnospira pectinoschiza*, further suggesting a potential ecological antagonism between low-gas-associated taxa and high-gas-producing taxa. Of particular note, *Faecalibacterium prausnitzii* was reduced in participants with flatulence and was significantly negatively correlated with gas production. Previous *in vitro* studies have also suggested that the highest gas-producing phenotype may be associated with a marked decrease in *Faecalibacterium* abundance.^40^ Thus, flatulence-associated dysbiosis is unlikely to be explained simply by an increase in gas-producing bacteria, but rather by the combined effects of expansion of potentially gas-producing taxa, depletion of strict anaerobic commensals, and weakening of butyrate-associated protective networks.

Consistent with these compositional alterations, the microbial functional profile and fecal metabolome of participants with flatulence further suggested that the intestinal microenvironment may shift from a homeostatic symbiotic state toward abnormal fermentation, oxidative stress, and low-grade inflammation. Under healthy conditions, the gut microbiota generally maintains a relatively complete capacity for nutrient biosynthesis, cofactor metabolism, carbohydrate uptake, and central carbon metabolism, which supports metabolic mutualism between the host and the microbiota. In contrast, the flatulence-associated microbiota showed enrichment of oxidative phosphorylation, reactive oxygen species-related pathways, and biofilm formation pathways associated with *Escherichia coli* and *Pseudomonas aeruginosa*, suggesting abnormal oxygen exposure and intensified oxidative stress in the intestinal microenvironment. For a highly anaerobic ecosystem such as the gut, enrichment of oxidative phosphorylation-related functions may indicate increased local oxygen exposure or a stronger respiratory metabolic advantage for facultative anaerobes. At the same time, enrichment of ROS-related pathways and pathogenic biofilm formation pathways suggests that some bacteria may be exposed to oxidative pressure and may acquire enhanced colonization adaptability. Such functional shifts may weaken the ecological niche of strict anaerobic commensals and create favorable conditions for the expansion of opportunistic pathogens or high-gas-producing taxa. The metabolomic data provided further chemical support for this interpretation. Increased levels of purine degradation products such as hypoxanthine are commonly associated with cellular stress, disturbed energy metabolism, and oxidative stress. The increase in indole derivatives such as 2-oxindole suggests a shift in tryptophan metabolism, which may be associated with abnormal protein fermentation and accumulation of intestinal irritant metabolites. Conversely, metabolites with potential anti-inflammatory, antioxidant, or mucosal protective properties, such as docosahexaenoic acid ethyl ester and 18-*β*-glycyrrhetinic acid, were reduced. In addition, butyrate was negatively correlated with gas production, further suggesting that insufficient butyrate-associated anti-inflammatory and barrier-protective networks may be linked to the high-gas phenotype and microbial instability. SCFA production is often accompanied by gas generation during intestinal fermentation. In the acetate-producing pathway, glucose is converted to two molecules of pyruvate through glycolysis; pyruvate is then decarboxylated to acetyl-CoA and CO₂ by pyruvate: ferredoxin oxidoreductase in association with reduced ferredoxin. Acetyl-CoA can subsequently enter either the acetate- or butyrate-producing branch.^41^ ^,^ ^42^ Although SCFAs have been shown to confer benefits in many disease contexts, they may also participate in IBS pathogenesis, and both fecal SCFA levels and the abundance of SCFA-associated bacteria are altered in patients with IBS.^43^ Tana et al.^44^ and Gargari et al.^45^ reported that fecal acetate, propionate, and total SCFA levels were significantly higher in patients with IBS than in controls. Higher acetate and propionate levels have been associated with worse gastrointestinal symptoms, reduced quality of life, and negative emotional status. Previous studies have also found enrichment of genes and pathways involved in carbohydrate metabolism in patients with IBS, which may increase anaerobic glycolysis and the production of CO₂, H₂, and CH₄, thereby enhancing intestinal distension and aggravating symptoms.^46^ Increased fecal calprotectin further supports, from the host side, the presence of low-grade inflammation or mucosal immune activation.

As a core strict anaerobic commensal and candidate next-generation probiotic in the human gut, *F. prausnitzii* has been reported to be depleted in multiple gastrointestinal diseases, including chronic constipation, celiac disease, IBS, and IBD. Its importance lies not only in its role as a major butyrate producer involved in host energy metabolism and anti-inflammatory homeostasis, but also in its potential contribution to maintaining a low-oxygen, reductive, and strictly anaerobic intestinal ecosystem through butyrate production. Metabolically, *F. prausnitzii* mainly relies on an acetate-dependent butyrate synthesis pathway: acetyl-CoA is converted to butyryl-CoA through enzymatic steps involving *AtoB/Thl*, *Hbd*, *Crt*, and *Bcd-EtfAB*, after which butyryl-CoA: acetate CoA-transferase *But* catalyzes CoA transfer with exogenous acetate to generate butyrate.^47^ This pathway enables *F. prausnitzii* to redirect acetate toward butyrate production and reduce fermentative pressure associated with acetate accumulation and H₂ generation. Unlike some hydrogenase-containing butyrate producers, *F. prausnitzii* does not rely on H₂ to drive butyrate production, and its metabolic products are less affected by H₂ concentration.^48^ Meanwhile, a considerable proportion of the carbon in *F. prausnitzii*-derived butyrate originates from exogenous acetate.^49^ Therefore, increasing the abundance of *F. prausnitzii* may help consume acetate, enhance butyrate production, reduce H₂ accumulation, and promote the recovery of the intestinal microenvironment from oxidative stress and abnormal fermentation toward a more stable anaerobic symbiotic state through the effects of butyrate on epithelial barrier function, immune regulation, and redox homeostasis. However, *F. prausnitzii* is not currently included among conventional edible bacterial species, and its strict anaerobic nature also limits direct formulation and application. Therefore, indirect enrichment of *F. prausnitzii* through diet or microbial interactions may be more feasible. For participants with flatulence, simple supplementation with conventional prebiotics carries potential risks, because fermentable substrates such as inulin and fructo-oligosaccharides may further increase colonic fermentation load and aggravate gas production.^50^ ^,^ ^51^ In contrast, bifidobacteria have metabolic features that are more suitable for this context. Bifidobacteria mainly metabolize pentoses and hexoses through the fructose-6-phosphate phosphoketolase pathway, also known as the bifid shunt, producing acetate and lactate but generally not gas.^52^ These metabolites can serve as cross-feeding substrates for butyrate-producing bacteria such as *F. prausnitzii*, thereby promoting butyrate production. Previous *in vitro* co-culture studies have demonstrated cross-feeding between *B. longum* and *F. prausnitzii,* ^53^ ^,^ ^54^ and clinical studies have shown that intervention with *B. longum* BB536 can increase the 16S rRNA gene copy number of *F. prausnitzii.* ^55^ Building on this evidence, CCFM1319 reduced gas production and promoted *F. prausnitzii* growth *in vitro*, and increased fecal *F. prausnitzii* absolute abundance while improving flatulence-related symptoms in a human intervention, providing in vivo evidence for targeted restoration of *F. prausnitzii* by *B. longum*. This strategy avoids the risk of gas production associated with directly increasing large amounts of fermentable substrates and suggests that precise restoration of low-gas-producing, butyrate-producing, and anti-inflammatory commensal networks through microbial interactions may represent a more ecologically selective approach for managing flatulence.

Overall, this study advances flatulence from the traditional concept of “excessive gas production or impaired gas evacuation” to a functional intestinal imbalance that can be interpreted and modulated through the gut microbiota. By identifying *F. prausnitzii* as a key target and further using *B. longum* CCFM1319 for indirect modulation, this study proposes a microbiota-interaction-based targeted intervention strategy, providing new evidence for the development of precision probiotics for flatulence and related functional gastrointestinal discomfort. Compared with simply increasing fermentable substrates or broadly modulating the microbiota, this strategy emphasizes the restoration of key commensals and their ecological functions, and therefore has promising translational potential. Future studies may further validate these microbial signatures and intervention effects in larger and more diverse populations, and evaluate their stability through long-term follow-up. In addition, the specific cross-feeding substrates, metabolic fluxes, and host responses through which CCFM1319 promotes *F. prausnitzii* expansion warrant further investigation using co-culture systems, stable isotope tracing, and targeted metabolomics. These studies will help refine flatulence intervention strategies centered on the restoration of key commensal bacteria.

Nevertheless, our study also has some limitations. First, although the flatulence symptom questionnaire was completed under the guidance and assessment of a gastroenterologist, the assessment was still based on self-reported symptoms; therefore, the influence of individual differences in symptom perception and reporting could not be completely excluded. Second, the sample sizes of the observational study and the randomized controlled intervention trial were sufficient to support the identification of flatulence-associated microbial features and the initial validation of the intervention effect, but future studies with larger sample sizes and more diverse populations will be helpful to further confirm these findings and enable more detailed subgroup analyzes. Finally, the *B. longum* CCFM1319 intervention was conducted over a relatively short period, which allowed us to preliminarily evaluate short-term symptom responses, microbiota changes, and safety. However, it remains unclear whether longer-term use would produce stronger effects, or whether the symptom relief observed in this study would persist after discontinuation of probiotic intake. Future long-term studies will be valuable for addressing these questions.

## Supplementary Material

Supplementary Material Supplementary materials.docx

## Data Availability

The 16S rRNA gene sequencing and metagenomic sequencing data generated in this study have been deposited in the China National GeneBank database under accession code CNP0009535 and CNP0009547.
