# Supplementary material for: Gut microbiota and metabolic alterations in participants with flatulence identify Faecalibacterium prausnitzii as a key microbial target for clinical intervention
Source: Gut Microbes. 2026 Sep 3;18(1):2728332. doi: 10.1080/19490976.2026.2728332 (PMC13556954; doi:10.1080/19490976.2026.2728332)
Supplement: Supplementary file 1 [file KGMI_A_2728332_SM7182.docx]

**Supplementary methods**

**Medium composition**

Gut microbiota medium (GMM) contained peptone, 2 g/L; yeast extract, 1 g/L; glucose, 0.4 g/L; cysteine, 0.5 g/L; cellobiose, 1 g/L; maltose, 1 g/L; fructose, 1 g/L; beef extract, 5 g/L; KH₂PO₄ solution, 100 mL/L (100 mmol/L); MgSO₄·7H₂O, 0.002 g/L; NaHCO₃, 0.4 g/L; NaCl, 0.08 g/L; CaCl₂ solution, 1 mL/L (0.80%, w/v); vitamin K₁, 1 mL/L; FeSO₄ solution, 1 mL/L (1.44 mmol/L); hemin-histidine solution, 1 mL/L (0.1%, w/v); Tween 80, 1 g/L; ATCC Vitamin Mix, 10 mL/L (0.1%, w/v); and ATCC Trace Mineral Mix, 10 mL/L (0.1%, w/v).

Simulation of colon chyme medium (SM) contained citrus pectin, 2 g/L; arabinogalactan, 2 g/L; inulin, 1 g/L; xylan, 2 g/L; soluble potato starch, 5 g/L; guar gum, 1 g/L; mucin, 4 g/L; acid-hydrolyzed casein, 3 g/L; yeast extract, 4.5 g/L; tryptone, 5 g/L; peptone water, 5 g/L; bile salts, 0.4 g/L; KH₂PO₄, 0.5 g/L; cysteine, 0.5 g/L; NaHCO₃, 1.5 g/L; KCl, 4.5 g/L; NaCl, 4.5 g/L; MgSO₄, 0.6 g/L; CaCl₂·2H₂O, 0.1 g/L; MnCl₂·4H₂O, 0.2 g/L; FeSO₄·7H₂O, 0.005 g/L; hemin chloride, 0.05 g/L; Tween 80, 1 g/L; and vitamin K₁, 1 mL/L.

M2GSC medium contained yeast extract, 5 g/L; casein peptone, 10 g/L; glucose, 5 g/L; fructose, 2 g/L; cellobiose, 2 g/L; NaHCO₃, 4 g/L; KH₂PO₄, 0.45 g/L; NaCl, 0.9 g/L; K₂HPO₄, 0.45 g/L; CaCl₂, 0.09 g/L; MgSO₄, 0.09 g/L; cysteine, 0.5 g/L; and clarified rumen fluid, 10 mL/L.

All media were sterilized at 115°C for 20 min. For GMM and SM, CaCl₂, vitamin K₁, FeSO₄, ATCC Vitamin Mix, and ATCC Trace Mineral Mix were sterilized by filtration through a 0.22-μm sterile filter and added to the autoclaved media after cooling.

Table S1. Baseline characteristics of in the observational cohort

| Baseline characteristics | Overall | Healthy | Flatulence | *P value* |
| --- | --- | --- | --- | --- |
| Number of participants | 76 | 32 | 44 |  |
| Female [n (%)] | 53（69.7） | 24（75.0） | 29（65.9） | 0.455 |
| Age (years) | 24.7 ± 6.27 | 22.8 ± 2.05 | 26.5 ± 7.66 | 0.076 |
| BMI（kg/m^2^） | 21.67 ± 2.54 | 21.13 ± 1.99 | 22.20 ± 2.89 | 0.169 |
| BSS | 3.95 ± 1.21 | 3.75 ± 0.88 | 4.09 ± 1.39 | 0.197 |

Data are presented as mean ± SD or n (%). BMI, body mass index; BSS, Bristol Stool Scale. Sex was compared using Fisher’s exact test; age, BMI and BSS were compared using the Mann-Whitney U test.

Table S2. Baseline characteristics of participants in the clinical intervention trial

| Baseline characteristics | Overall | Probiotic | Placebo | *P value* |
| --- | --- | --- | --- | --- |
| Number of participants | 46 | 30 | 16 |  |
| Female [n (%)] | 27（58.7） | 20（66.7） | 7（43.8） | 0.209 |
| Age (years) | 31.56 ± 13.61 | 31.17 ± 12.77 | 33.50 ± 14.97 | 0.611 |
| BMI（kg/m^2^） | 23.27 ± 3.50 | 23.67 ± 3.87 | 22.49 ± 2.64 | 0.237 |
| BSS | 4.38 ± 1.37 | 4.43 ± 1.41 | 4.27 ± 1.34 | 0.710 |

Data are presented as mean ± SD or n (%). BMI, body mass index; BSS, Bristol Stool Scale. Sex was compared using Fisher’s exact test; age, BMI and BSS were compared using the Mann-Whitney U test.

Table S3. Significantly different fecal metabolites between participants with flatulence and healthy controls

| Fecal metabolite | log2(FC) | -log10(pVal) | Legend |
| --- | --- | --- | --- |
| (+/-)12(13)-DiHOME | -1.33377 | 17.59399 | Sig_Down |
| (1R,2R,6R,9R)-2,11,11-trimethyl-3-oxotricyclo[4.3.2.0]undecane-9-carboxylic acid | -1.02991 | 7.590743 | Sig_Down |
| (2E,4E)-N-(2-methylpropyl)deca-2,4-dienamide | -1.19796 | 4.024761 | Sig_Down |
| (3S)-3-Methyl-5-[(1S,8aR)-2,5,5,8a-tetramethyl-4-oxo-1,4,4a,5,6,7,8,8a-octahydro-1-naphthalenyl]pentanoic acid | -1.46638 | 13.99663 | Sig_Down |
| (5ξ,9ξ,16ξ)-17-Hydroxykauran-19-oic acid | -1.05245 | 4.309388 | Sig_Down |
| 18-β-Glycyrrhetinic acid | -1.34942 | 3.01112 | Sig_Down |
| 2,4-Dimethylbenzaldehyde | -1.27408 | 30.35674 | Sig_Down |
| 2-Amino-1,3,4-octadecanetriol | -3.17462 | 1.77705 | Sig_Down |
| 3-Methyl-5-(5,5,8a-trimethyl-2-methylene-7-oxodecahydro-1-naphthalenyl)pentyl acetate | -1.28254 | 3.480015 | Sig_Down |
| Cholest-4-en-3-one | -3.37295 | 2.579186 | Sig_Down |
| Docosahexaenoic acid ethyl ester | -2.34168 | 10.933 | Sig_Down |
| L-(+)-Arginine | -1.64372 | 2.177969 | Sig_Down |
| N6,N6,N6-Trimethyl-L-lysine | -1.33858 | 1.868445 | Sig_Down |
| NP-016582-3 | -1.1788 | 3.780258 | Sig_Down |
| 16-Hydroxyhexadecanoic acid | 3.143148 | 3.19488 | Sig_Up |
| 1-Methylxanthine | 1.391768 | 3.082038 | Sig_Up |
| 2'-Deoxyadenosine | 1.62784 | 4.188908 | Sig_Up |
| 2'-Deoxyguanosine | 1.124196 | 2.627456 | Sig_Up |
| 2-Hydroxycaproic acid | 2.776904 | 1.348964 | Sig_Up |
| 2-Hydroxyphenylalanine | 2.823342 | 4.502269 | Sig_Up |
| 2-Oxindole | 1.101516 | 1.355266 | Sig_Up |
| 3-Phenyllactic acid | 4.010601 | 5.741818 | Sig_Up |
| 4-Dodecylbenzenesulfonic acid | 1.148544 | 1.773606 | Sig_Up |
| 4-Pyridoxic acid | 4.80333 | 3.405873 | Sig_Up |
| Acetophenone | 1.30772 | 3.699992 | Sig_Up |
| Berberine | 5.382806 | 5.174139 | Sig_Up |
| Bis(4-ethylbenzylidene)sorbitol | 2.407869 | 11.68715 | Sig_Up |

Continued Table S3

| Fecal metabolite | log2(FC) | -log10(pVal) | Legend |
| --- | --- | --- | --- |
| D-(-)-Arabinose | 1.326422 | 1.683401 | Sig_Up |
| D(+)-Phenyllactic acid | 2.0719 | 1.308397 | Sig_Up |
| D-(+)-Tryptophan | 2.489003 | 7.346305 | Sig_Up |
| DL-4-Hydroxyphenyllactic acid | 4.16784 | 6.586298 | Sig_Up |
| DL-Lysine | 1.821588 | 2.224528 | Sig_Up |
| DL-Tryptophan | 3.004987 | 4.064508 | Sig_Up |
| DL-β-Leucine | 2.735002 | 1.808465 | Sig_Up |
| Dodecanedioic acid | 2.435949 | 2.661046 | Sig_Up |
| Glycyl-L-leucine | 1.815411 | 5.149256 | Sig_Up |
| Guanine | 2.081169 | 3.164887 | Sig_Up |
| Hydrocinnamic acid | 4.083043 | 2.294838 | Sig_Up |
| Hypoxanthine | 1.663162 | 7.41384 | Sig_Up |
| Indole-3-acrylic acid | 2.774882 | 2.693253 | Sig_Up |
| Indole-3-lactic acid | 2.10286 | 1.59503 | Sig_Up |
| Leucylproline | 2.584506 | 4.018145 | Sig_Up |
| L-Glutamic acid | 6.082958 | 4.686006 | Sig_Up |
| L-Norleucine | 3.178906 | 2.916999 | Sig_Up |
| L-Phenylalanine | 3.742761 | 2.66352 | Sig_Up |
| L-Tyrosine | 3.838548 | 5.673541 | Sig_Up |
| Myristic acid | 2.776062 | 1.982257 | Sig_Up |
| N-Acetyl-D-alloisoleucine | 3.686949 | 6.490717 | Sig_Up |
| N-Acetyl-DL-tryptophan | 1.387528 | 2.065052 | Sig_Up |
| N-Acetyl-L-phenylalanine | 1.90246 | 4.591099 | Sig_Up |
| Pantothenic acid | 4.481945 | 4.87913 | Sig_Up |
| Pentadecanoic acid | 2.760923 | 3.698016 | Sig_Up |
| Thymine | 2.262373 | 3.080734 | Sig_Up |
| trans-Petroselinic acid | 3.142985 | 2.912787 | Sig_Up |
| Valeric acid | 3.311212 | 1.310336 | Sig_Up |
| δ-Valerolactam | 3.232384 | 4.519059 | Sig_Up |

**Figure S1. Participant flow diagram for the cross-sectional observational study**

**Figure S2. Participant flow diagram for the randomized, double-blind, placebo-controlled intervention trial with *B. longum* CCFM1319.**

**Figure S3. Effects of disease status and sampling time on gut microbial structure and taxonomic composition of the two groups**

(a) Adonis multivariate analysis evaluating the contribution of disease status and sampling time to variation in gut microbial β-diversity. Bars indicate the proportion of community variation explained by each factor (R²).

(b–d) Stacked bar plots showing the taxonomic composition of the gut microbiota in healthy controls and participants with flatulence at the phylum (b), family (c), and genus (d) levels. Each bar represents the mean relative abundance of major taxa in each group.

**Figure S4. Species significantly correlated with in vitro gas production under GMM and SM fermentation conditions.**

(a) Genus-level microbial composition of samples ranked from low to high according to *in vitro* gas production. Black dots above the stacked bars indicate gas production for each sample, and the stacked bar plots below show the corresponding genus-level relative abundance. The left panel shows fermentation under GMM conditions, and the right panel shows fermentation under SM conditions.

(b) Spearman correlation networks of species significantly associated with gas production. Nodes represent species significantly correlated with gas production, and edges indicate interspecies correlations. Red edges indicate positive correlations, whereas dark gray edges indicate negative correlations. Node size represents relative abundance or network connectivity. The upper panel shows fermentation under GMM conditions, and the lower panel shows fermentation under SM conditions.

**Figure S5. Differential metabolite profiles after in vitro replenishment with *F. prausnitzii***

The heatmap shows changes in the relative abundance of differential metabolites in the control group, *F. prausnitzii* A2-165 replenishment group, and CCFM1204 replenishment group after *in vitro* fermentation.

**Figure S6 Correlations between the relative abundance of *Bifidobacterium* species and *Faecalibacterium prausnitzii* in fecal samples from Cross-sectional observational study.**

(a) Spearman correlation analysis between the relative abundance of *F. prausnitzii* and *B. longum*

(b) Spearman correlation analysis between the relative abundance of *F. prausnitzii* and *B. pseudocatenulatum*.

Red points represent individual samples. Black lines indicate linear regression fits, and grey shaded areas indicate 95% confidence intervals.

**Figure S7. Strain stability, baseline characteristics, and GSRS scores in the *B. longum* CCFM1319 clinical intervention trial**

(a) Changes in the viable count of *B. longum* CCFM1319 used in the clinical intervention.

(b) Baseline flatulence-related symptom scores in the CCFM1319 and placebo groups, including flatulence, bloating, abdominal distension, borborygmi, and gastrointestinal comfort.

(c) Changes in total Gastrointestinal Symptom Rating Scale (GSRS) scores before and after intervention in the CCFM1319 and placebo groups.
